# Autozygome and high throughput confirmation of disease genes candidacy

**DOI:** 10.1038/s41436-018-0138-x

**Published:** 2018-09-21

**Authors:** Sateesh Maddirevula, Fatema Alzahrani, Mohammed Al-Owain, Mohammad A. Al Muhaizea, Husam R. Kayyali, Amal AlHashem, Zuhair Rahbeeni, Maha Al-Otaibi, Hamad I. Alzaidan, Ameera Balobaid, Heba Y. El Khashab, Dalal K. Bubshait, Maha Faden, Suad Al Yamani, Omar Dabbagh, Mariam Al-Mureikhi, Abdulla Al Jasser, Hessa S. Alsaif, Iram Alluhaydan, Mohammed Zain Seidahmed, Bashair Hamza Alabbasi, Ibrahim Almogarri, Wesam Kurdi, Hana Akleh, Alya Qari, Saeed M. Al Tala, Suzan Alhomaidi, Amal Y. Kentab, Mustafa A. Salih, Aziza Chedrawi, Seham Alameer, Brahim Tabarki, Hanan E. Shamseldin, Nisha Patel, Niema Ibrahim, Firdous Abdulwahab, Menasria Samira, Ewa Goljan, Mohamed Abouelhoda, Brian F. Meyer, Mais Hashem, Ranad Shaheen, Saad AlShahwan, Majid Alfadhel, Tawfeg Ben-Omran, Mohammad M. Al-Qattan, Dorota Monies, Fowzan S. Alkuraya

**Affiliations:** 1 Department of Genetics, King Faisal Specialist Hospital and Research Center, Riyadh, Saudi Arabia; 2 Department of Medical Genetics, King Faisal Specialist Hospital and Research Center, Riyadh, Saudi Arabia; 3 College of Medicine, Alfaisal University, Riyadh, Saudi Arabia; 4 Department of Neurosciences, King Faisal Specialist Hospital and Research Center, Riyadh, Saudi Arabia; 5 Department of Pediatrics, King Faisal Specialist hospital and Research Center, Jeddah, Saudi Arabia; 6 Department of Pediatrics, Prince Sultan Military Medical City, Riyadh, Saudi Arabia; 7 Genetic Unit, Children’s Hospital, King Saud Medical City, Riyadh, Saudi Arabia; 8 Department of Pediatrics, Children’s Hospital, Ain Shams University, Cairo, Egypt; 9 Department of Pediatrics, Dr. Suliman Al Habib Medical Group, Riyadh, Saudi Arabia; 10 Department of Pediatrics, College of Medicine, Imam Abdulrahman Bin Faisal University, Dammam, Saudi Arabia; 11 Section of Clinical and Metabolic Genetics, Department of Pediatrics, Hamad Medical Corporation, Doha, Doha, Qatar; 12 Genetics Division, Department of Pediatrics, King Saud Bin Abdulaziz University for Health Sciences, King Abdulaziz Medical City, Riyadh, Saudi Arabia; 13 Department of Pediatrics, Security Forces Hospital, Riyadh, Saudi Arabia; 14 Department of Pediatrics, King Faisal Specialist Hospital and Research Center, Riyadh, Saudi Arabia; 15 Department of Obstetrics and Gynecology, King Faisal Specialist Hospital and Research Center, Riyadh, Saudi Arabia; 16 Department of Neuroscience, King Faisal Specialist Hospital and Research Center, Riyadh, Saudi Arabia; 17 Department of Pediatrics, Armed Forces Hospital SR, Khamis Mushayt, Saudi Arabia; 18 Department of Pediatrics, College of Medicine, King Saud University, Riyadh, Saudi Arabia; 19 Department of pediatrics, King Abdulaziz Medical City, Jeddah, Saudi Arabia; 20 Saudi Human Genome Program, King Abdulaziz City for Science and Technology, Riyadh, Saudi Arabia; 21 Medical Genetic Division, Department of Pediatrics, King Abdullah International Medical Research Centre, King Saud bin Abdulaziz University for Health Sciences, King Abdulaziz Medical City, Riyadh, Saudi Arabia; 22 Department of Surgery, King Faisal Specialist Hospital and Research Center, Riyadh, Saudi Arabia

**Keywords:** ACMG guidelines, variant interpretation, candidate genes

## Abstract

**Purpose:**

Establishing links between Mendelian phenotypes and genes enables the proper interpretation of variants therein. Autozygome, a rich source of homozygous variants, has been successfully utilized for the high throughput identification of novel autosomal recessive disease genes. Here, we highlight the utility of the autozygome for the high throughput confirmation of previously published tentative links to diseases.

**Methods:**

Autozygome and exome analysis of patients with suspected Mendelian phenotypes. All variants were classified according to the American College of Medical Genetics and Genomics guidelines.

**Results:**

We highlight 30 published candidate genes (*ACTL6B*, *ADAM22*, *AGTPBP1*, *APC*, *C12orf4*, *C3orf17 (NEPRO)*, *CENPF*, *CNPY3*, *COL27A1*, *DMBX1*, *FUT8*, *GOLGA2*, *KIAA0556*, *LENG8*, *MCIDAS*, *MTMR9*, *MYH11*, *QRSL1*, *RUBCN*, *SLC25A42*, *SLC9A1*, *TBXT*, *TFG*, *THUMPD1*, *TRAF3IP2*, *UFC1*, *UFM1*, *WDR81*, *XRCC2*, *ZAK*) in which we identified homozygous likely deleterious variants in patients with compatible phenotypes. We also identified homozygous likely deleterious variants in 18 published candidate genes (*ABCA2*, *ARL6IP1*, *ATP8A2*, *CDK9*, *CNKSR1*, *DGAT1*, *DMXL2*, *GEMIN4*, *HCN2*, *HCRT*, *MYO9A*, *PARS2*, *PLOD3*, *PREPL*, *SCLT1*, *STX3*, *TXNRD2*, *WIPI2*) although the associated phenotypes are sufficiently different from the original reports that they represent phenotypic expansion or potentially distinct allelic disorders.

**Conclusions:**

Our results should facilitate the timely relabeling of these candidate disease genes in relevant databases to improve the yield of clinical genomic sequencing.

## Introduction

Mendelian genetics has seen some of the most successful applications of genomic medicine. The often-clear departure of Mendelian phenotypes from the population average, the high confidence with which these phenotypes are attributed to single variants, and the rare and deleterious nature that often characterizes these variants are important contributing factors.^1^ A major concern in Mendelian genetics, however, is that there are potentially thousands of genes that have yet to be linked to Mendelian phenotypes, a critical gap that greatly impedes the full realization of the benefits of clinical genomic sequencing. For example, the commonly cited 25% clinical sensitivity of genomic sequencing in the setting of Mendelian diseases appears to correlate with the extent to which the genetic heterogeneity of a particular phenotype had been captured by prior research.^2^ Similarly, recent papers suggest that revisiting exome sequencing based on newly annotated disease genes can boost the yield.^3–7^ Thus, it remains a priority in the field of clinical laboratory genetics to have all genes with links to Mendelian phenotypes established in databases that are commonly used in the variant annotation pipeline, e.g., OMIM. The American College of Medical Genetics and Genomics (ACMG) guidelines for variant interpretation clearly state that variants that would otherwise qualify as likely pathogenic or even pathogenic cannot be reported as such in genes with no established link to disease, and as such these variants should be reported, if at all, as variants of unknown significance.^8^

The International Rare Diseases Research Consortium (IRDiRC) states in its vision that all patients with genetic disorders should receive a molecular diagnosis by the year 2020 (ref.^9^). It is more realistic, however, to hope that the entire Mendeliome will be curated by that date. This optimism is fueled by the remarkable rate at which novel disease genes are identified, thanks in large part to the democratization of genomic sequencing that made it possible for the first time for patients to have their genomes sequenced clinically thus bypassing the bottleneck of testing by research labs with specific interest in their phenotypes.^10^ Notwithstanding the concern that the pace of such discoveries may have slowed, a new bottleneck has quickly emerged in that as we tackle diseases of exceeding rarity, we are more likely to face the “*n* of one” challenge.^11^ While publishing candidate genes based on single pathogenic variants is often insufficient to establish links to diseases unambiguously, such publications are often the impetus for follow-up confirmatory papers. The percentage of novel disease genes that are subsequently confirmed when their initially published candidacy was based on single mutational event is unknown. However, this percentage likely depends on the strength of other lines of supporting evidence in the original publication, e.g., positional mapping, segregation, functional analysis, etc. For example, of the 66 candidate genes we published based on single pathogenic variants that are also supported by compelling positional mapping data, i.e., a single autozygous locus, 54 were subsequently confirmed and none was challenged or disputed (unpublished data).

The power of autozygosity, however, is not limited to the initial mapping and reporting of novel disease genes. As a vast source of homozygous variants, the autozygome may also serve as an efficient tool for the high throughput confirmation of disease genes candidacy by supplying additional deleterious variants in those gene. Even when the same previously reported variants are encountered in "unrelated" patients with the same haplotypes, there is further strengthening of the previously published positional mapping data and thus the candidacy of the respective genes. In this study, the largest of its kind, we implemented this approach to support the candidacy of 48 previously reported novel candidate disease genes.

## Materials and methods

### Human subjects

Subjects were either consented as part of an institutional review board–approved research protocol (in the case of research-grade exomes, KFSHR RAC 2121053, 2140016, 2080006, 2070023) or a standard clinical exome consent (in the case of clinical exomes). Phenotypes were provided by in-depth clinical evaluation by the participating clinicians. Where applicable, additional consent to show identifiable patient information were also obtained.

### Variant identification

We searched our database of 799 research and 1750 clinical exomes in search of homozygous variants that otherwise qualify for pathogenic or likely pathogenic classification according to the ACMG guidelines but could not be classified as such because the involved genes only have tentative links to diseases. We defined genes as having tentative links if they belonged to one of the following three categories:

a. genes with previously reported pathogenic variants in the context of a Mendelian phenotype but remain unlisted as disease genes in OMIM

b. genes that are listed in OMIM as disease genes but with “?” mark indicating insufficient evidence

c. genes that are listed in OMIM as diseases genes without “?” mark but the evidence is based on a single study

The autozygome was defined by the entire set of autozygous blocks per individual.^12^ Determining whether the variant resides within the autozygome was made directly from the exome data as described in detail elsewhere.^13^

### Potential confirmation and phenotypic definition

We considered two categories for the potential confirmation of candidacy. Category 1 is when a homozygous variant other than the one previously reported is identified. Category 2 is when the previously reported candidacy was based on a single homozygous variant and we identified the same variant on the same haplotype block in an individual who is not part of the originally reported sibship. For defining the phenotypic overlap, we considered three classes:

Class 1: The phenotype is identical or very similar to that previously reported.

Class 2: The phenotype is similar but involves additional features, i.e., phenotypic expansion.

Class 3: The phenotype was sufficiently different to qualify as a potentially different clinical presentation, i.e., distinct allelic disorder. We opted to consider this possibility rather than challenge or dispute the previous reports to accommodate the increasing appreciation of variable phenotypic expression of Mendelian genes.^14^

## Results

### Potential confirmation of 48 previously reported novel disease genes

Supplementary Table 1 summarizes the 52 cases (44 reported here for the first time and their full clinical phenotypes are described in Table S1) whose autozygome contained homozygous variants that argue in support of previously reported tentative disease–gene links. These variants were mostly (*n* = 32, ~66%) truncating thus potentially rendering the affected individuals natural knockouts for the respective genes, and as such confer further confidence in the previously reported links (especially when such links were based on hypomorphic missense variants).^15^ Examples include *ABCA2*, *HCN2*, *MYO9A*, *QRSL1*, and *THUMPD1*. Missense variants were similarly carefully chosen to be likely deleterious based on novelty or extreme rarity (minor allele frequency [MAF] < 0.001 in gnomAD and local exome database), and consistent deleterious in silico prediction by SIFT, PolyPhen, and CADD (Supplementary Table 1).

The overwhelming majority of confirmation events were based on category 1, i.e., independent pathogenic variant events. We only encountered five instances of category 2, i.e., same previously reported variant on a similar haplotype, and these involved *CDK9*, *GEMIN4*, *C3orf17*, *WDR81*, *SCLT1*, and *UFC1*. We have previously reported a single pathogenic variant each in *CDK9*, *GEMIN4*, *C3orf17*, *WDR81*, *SCLT1*, and *UFC1* in patients with CHARGE-like presentation (coloboma, choanal atresia, and urogenital malformations),^16^ syndromic cataract (cataract with static encephalopathy),^17^ Ehlers–Danlos-like syndrome,^16,18^ congenital hydrocephalus (another variant in *WDR81* was also reported),^19^ oral–facial–digital syndrome (OFD),^20^ and epileptic encephalopathy,^13^ respectively. We have identified the same founder variants in individuals with the same phenotypes that we previously attributed to these genes. In addition, it is worth highlighting that in two instances, we report recessive pathogenic variants  in genes previously proposed as candidate genes based on monoallelic pathogenic variants: *HCRT* and *WIPI2*.

### Defining the phenotypic spectrum of previously reported candidate genes

**Fig. 1:**
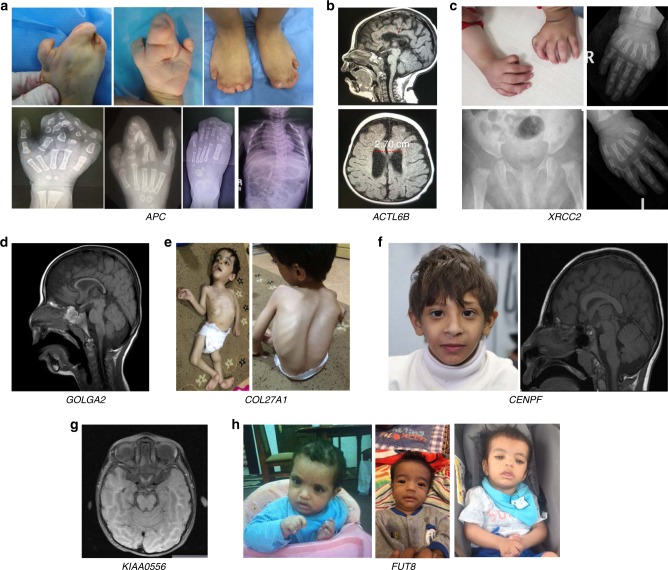
**Representative images of cases with class 1 phenotypes.** **a** Panel of clinical images of the proband with *APC*-related Cenani–Lenz syndrome showing the classic bony syndactyly with loss of the normal configuration of the phalanges. **b** Brain magnetic resonance image (MRI) of the proband with pathogenic variant in *ACTL6B* showing agenesis of corpus callosum and mild ventricular dilatation (2.70 cm). **c** Typical skeletal findings in the proband with *XRCC2*-related Fanconi anemia showing hypoplastic thumbs and dysplastic hips. **d** Brain MRI image showing thin but complete corpus callosum in the proband with *GOLGA2* pathogenic variant. **e** Clinical images of the proband with Steel syndrome (pathogenic variant in *COL27A1*) showing characteristic facial and skeletal appearance (elongated face with severe scoliosis). **f** Facial photograph and MRI brain of the proband with pathogenic variant in *CENPF* showing small head, relatively large ears, epicanthal folds, blue-tinged sclerae, prominent nose, thin upper lip, and small chin. MRI revealed reduced white matter volume and marginal pachygyria. **g** Brain MRI image showing mild molar tooth sign in the proband with KIAA0556 pathogenic variant. **h** Facial photographs of proband with *FUT8* pathogenic variant showing coarse face with synophrys, hypertelorism, sparse eye-lashes, low set ears, thick inverted V-shaped lips.

**Fig. 2:**
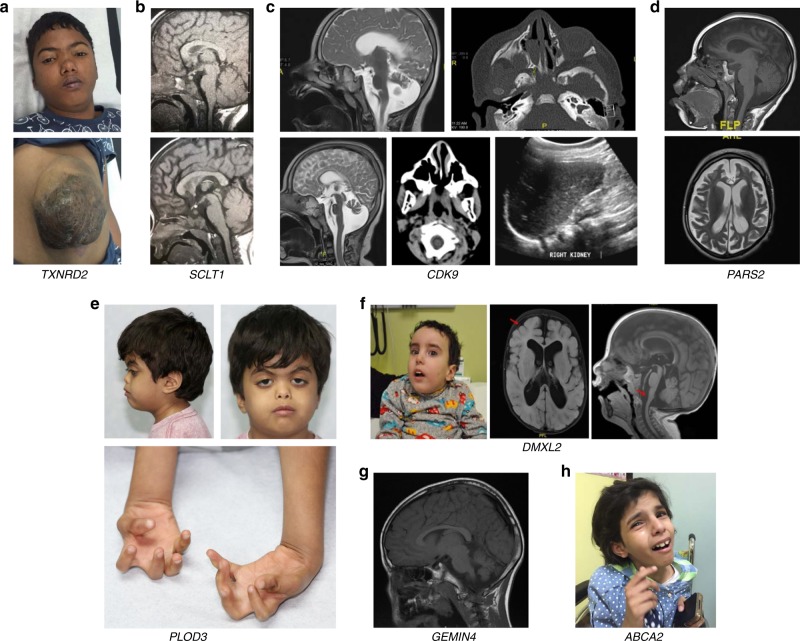
**Representative images of cases with class 2 and 3 phenotypes.** **a** Clinical images of the proband with *TXNRD2* pathogenic variant showing dysmorphic facies and a large umbilical hernia. **b** The two siblings with *SCLT1* pathogenic variant showing absence of the anterior lobe of pituitary gland with the evidence of ectopia of the posterior pituitary gland and thalamic hamartoma. **c** Three families are with *CDK9*-related CHARGE-like phenotype showing cerebellar atrophy, unilateral choanal atresia, and dysplastic atrophic kidney. **d** Brain magnetic resonance image (MRI) of proband with pathogenic variant in *PARS2* showing severe cerebral volume loss indicating severe brain atrophy, thinning of the corpus callosum, and severe cerebral volume loss in the supratentorial and infratentorial area. **e** Clinical images of the proband with *PLOD3* pathogenic variant showing dysmorphic facies (prominent forehead, short and flat nose, ptosis with compensatory arching of eyebrows, posteriorly rotated low set ears) and hand contractures. **f** Facial features and MRI images of proband with *DMXL2* pathogenic variant represent long face, high forehead, short philtrum, low set ears, and moderate degree of cerebral and brainstem atrophy. **g** Brain MRI image showing small atrophic cerebellum with prominence of the posterior fossa cerebrospinal fluid (CSF) spaces in the proband with *GEMIN4* pathogenic variant. **h** Facial pictures of index (16DG0071) subject with *ABCA2* showing apparent lack of gross dysmorphism.

We have encountered 30 genes with mutational events that represent no clear phenotypic expansion, i.e., class 1 (Supplementary Table 1 and Fig. 1). We have also encountered 17 instances (15 genes) of phenotypic expansion, i.e., class 2 (Supplementary Table 1 and Fig. 2). A few of these are worth highlighting. For example, the tentative OMIM phenotype for *ARL6IP1* is spastic paraplegia based on a single homozygous frameshift variant. However, the patient in whom we identified a homozygous truncating variant presented with severe neonatal hypotonia. Similarly, the only reported patient with the OMIM phenotype of cerebellar ataxia, mental retardation, and dysequilibrium syndrome had a single missense variant in *ATP8A2* whereas the patient we present with a homozygous truncating variant has physical and cognitive delay, failure to thrive, vision impairment, hypotonia, eczema and leukodystrophy. Another interesting example is lysyl hydroxylase 3 deficiency, which is listed in OMIM based on a single compound heterozygous pathogenic variant. While our patient with a homozygous truncating variant similarly has significant connective tissue involvement, his presentation seems to expand the phenotype by featuring microcephaly, and ptosis (Fig. 2). Similarly, our patient 17-3959 with a homozygous truncating variant in *TXNRD2* had a syndromic manifestation characterized by low cortisol, intellectual disability, epilepsy, dysmorphic features, truncus arteriosus, and omphalocele (Fig. 2), unlike isolated glucocorticoid deficiency that was reported in OMIM. Case 17DG1005 is rather unusual in that it represents a phenotypic “contraction” rather than expansion. In 2015, we reported the identification of *SCLT1* along with *TBC1D32/C6orf170* as two novel genes for OFD IX based on two families with severe midline defects including cleft and hypopituitarism, in addition to microphthalmia and polydactyly.^20^ The same *SCLT1* founder mutation was later identified in a child with a milder form of intellectual disability, obesity, coloboma, renal failure, and subsequent renal transplant, retinopathy, midline cleft, epilepsy, atopy, panhypopituitarism, and undescended left testicle.^21^ However, case 17DG1005 and his sibling both presented with isolated panhypopituitarism and hypothalamic hamartoma with no associated syndromic features of OFD or other ciliopathies (Fig. 2). *PARS2* was previously implicated in a mitochondrial phenotype featuring neurodegeneration and liver involvement (Alpers syndrome) based on compound heterozygous pathogenic variants.^22–24^ Interestingly, in one family we show that homozygosity for a variant in *PARS2* (NM_152268.3:c.283G>A:p.[Val95Ile]), previously reported only in *trans* with another allele, fully segregated with global developmental delay, epilepsy, and brain atrophy but without lactic acidosis or renal or liver involvement (Fig. 2).

In class 3, the phenotypes observed are sufficiently different from those previously reported that we argue these may represent distinct allelic disorders (Supplementary Table 1, Fig. 2). These include two families with global developmental delay and intellectual disability (Fig. 2) that fully segregated with homozygous truncating variants in *ABCA2* (NM_212533.2:c.740dupT:p.[Gly248Argfs*38]) and (NM_212533.2:c.1027C>T:p.[Gln343*]), which is distinct from the amyotrophic lateral sclerosis reported by Steinberg et al.^25^ We also found that *DMXL2* harbors a homozygous truncating variant (NM_001174117.1:c.4349_4350insTTACATGA:p.[Glu1450Aspfs*23]) in a child with intellectual disability, epilepsy, macrocephaly, and dysmorphism (Fig. 2), in contrast to the multiple endocrinopathies reported by Tata et al. based on a single pathogenic variant.^26^ Finally, *WIPI2* was reported as a candidate gene for cerebral palsy based on a single de novo pathogenic variant by McMichael et al.^27^ However, our patient with a homozygous truncating variant in this gene presented with hypotonia, failure to thrive, nystagmus, elevated transaminases, and raised α-fetoprotein.

## Discussion

The growing demand for clinical sequencing calls for an acceleration in curating the Mendeliome. Variants in the Mendeliome are not only helpful in diagnosis and management, but also in reproductive planning and even in identifying drug targets for common diseases.^1,28,29^ It is likely that in the near future, the overwhelming majority of patients with Mendelian diseases will have their causal variants captured by genome sequencing. However, the confidence with which such variants are called as such will depend on whether the genes in which these variants are identified had been linked to the patient’s phenotype. Sharing of variants across different cohorts will likely accelerate successful matching.^30^ However, it is likely that postpublication matchmaking will remain an important method for the independent validation of candidate genes. In this study, we attempted to maximize the power of postpublication matchmaking by reporting a large group of likely deleterious homozygous variants that were facilitated by the phenomenon of autozygosity in genes previously published as candidates. While there is value in dedicating a separate publication to each of these confirmatory reports, we believe that their grouping will provide a more timely communication of results.

Not surprisingly, the phenotypes we report in each of the confirmatory cases range from identical to quite distinct from those previously reported. We have previously shown how recessive human knockouts can present with surprisingly different phenotypes compared with those with monoallelic pathogenic variants, and this study adds several examples of this phenomenon, e.g., *MYH11*-related megacystis.^14^ However, we also note that in several other cases, marked phenotypic heterogeneity was noted even when compared with previously reported cases with biallelic pathogenic variants. Although we have looked carefully for additional variants that may have resulted in these unusual phenotypes (i.e., multilocus phenotypes, also referred to as dual molecular diagnosis), none were identified in the cases presented. Thus, it seems likely that these cases may represent true phenotypic expansion, especially considering the very limited cases that had been reported. Obviously, additional cases in the future will be needed to delineate the true phenotypic spectrum of the respective genes.

The ClinGen consortium has recently published an attempt to standardize the establishment of disease–gene links.^31^ This will likely be one of many steps going forward as we grapple with such issues as extreme phenotypic heterogeneity and exceedingly rare diseases. The uncertainty that surrounds many disease–gene links can be seen in OMIM disease genes listings, which sometimes include genes with single spathogenic variants but not genes with several reported pathogenic variants. While it is agreed that two independent pathogenic variants are not necessarily sufficient to unambiguously link a gene to a specific phenotype, this remains one of the best confirmatory methods. On the other hand, compelling positional mapping data may sometimes be the only possible means of confirmation especially for phenotypes that are allele-specific. For example, although only one single missense variant in *ADAT3* has been reported, it has emerged as the single most common cause of nonsyndromic autosomal recessive intellectual disability in Arabia such that there is now little doubt about the disease–gene link.^32–35^ It is for this reason that we opted to include in this study, as another confirmatory method, cases with similar variants to those previously reported if they corroborate the positional mapping evidence even as we acknowledge the possibility of being in linkage disequilibrium with the actual causal variants. Examples of disorders we were able to confirm through this approach in this study are *TBXT*-related myelomeningocele,^36^ *APC*-related Cenani–Lenz syndrome,^37^ and *XRCC2*-related Fanconi anemia.^38^

There is now a growing appreciation that deleterious recessive variants may express phenotypically in different ways depending on whether they are encountered in homozygosity or in the compound heterozygous state with other variants. Thus, the autozygome provides a unique opportunity to unmask the phenotypic expression of variants that had only been reported as compound heterozygous.^39^ For example, we show here that one *PARS2* allele that was reported to cause Alpers syndrome when compound heterozygous only causes nonspecific global developmental delay and brain atrophy when homozygous but with no evidence of lactic acidosis or liver involvement when homozygous. With its potential to render many previously reported variants as homozygous, the autozygome can be very powerful in revealing important phenotypic aspects of alleles, which may not be appreciated otherwise.

In summary, we present the largest confirmatory cohort to date in the setting of genes with tentative links to Mendelian phenotypes, all based on autozygome-related homozygosity for likely deleterious variants. This effort will complement others to accelerate the high confidence mapping of the Mendeliome. The resulting phenotypes, whether overlapping or distinct, will add to our understanding of the medical relevance of the respective genes in different contexts, and will further enhance the accuracy of variant interpretation.

## Electronic supplementary material

Supplementary Table S1

Supplementary Table 1
