# Supplementary material for: Autozygome and high throughput confirmation of disease genes candidacy
Source: Genet Med. 2018 Sep 21;21(3):736–42. doi: 10.1038/s41436-018-0138-x (PMC6752307; doi:10.1038/s41436-018-0138-x)
Supplement: Supplementary file 1 — Supplementary Table S1 [file 41436_2018_138_MOESM1_ESM.docx]

**Table S1.** Detailed clinical manifestation of all probands.

| 16-2987 | *ABCA2*  NM_212533.2:c.740dupT:p.(Gly248Argfs*38) | A 10-year-old Saudi boy with history of global developmental delay and epilepsy. He was born full term. His birth weight was 3.5 kg. He had multiple admissions in the first year of life for fever, diarrhea, vomiting and seizures. He has global developmental delay. Parents are distantly related. He has one brother and three sisters, all of whom are healthy. He has mild dysmorphic features with prominent forehead and somewhat deep-set eyes. He has no birth marks or hepatosplenomegaly. Pupils, extraocular muscles, facial, and tongue movements are all symmetrical and normal. He has diffuse hypotonia and weakness but moves all his extremities against gravity. He localized to pain upon stimulation throughout. Brain MRI was unremarkable. |
| --- | --- | --- |
| 16DG0071 | *ABCA2* NM_212533.2:c.1027C>T:p.(Gln343*) | A 13 years old girl born to G1P0 19 yr old mother, pregnancy was uncomplicated. She developed epilepsy at 9 months of age with concomitant developmental delay. She is short (131.5 cm), microcephalic (50 cm). She had significant internal rotation of the hips especially the left. MRI showed small pituitary gland. Parents are first cousins and have two healthy daughters. |
| 17-1447 | *ACTL6B* NM_016188.4:c.999T>A:p.(Cys333*) | A 13 months old girl born to consanguineous parents. She presented with hyperekplexia and global developmental delay. MRI shown agenesis of corpus collosum with associated with mild ventricular dilatation, mild atrophic changes, minimally simplified gyral pattern with prominence of sulci, mild posterior colpocephaly. High lactate peak by MRS. There is history of a similarly affected brother who died at 2 months of age. |
| 15DG1266 | *ADAM22*  NM_016351.4:c.2578C>T:p.(Arg860*) | An 18-year-old male, a product of consanguineous marriage, born at term via spontaneous vaginal delivery without postnatal complications with birth weight of 3.5 Kg. His first seizure started at age 5 months as left-sided focal seizure (mainly upper limb) with left eye blinking, and facial twitching, that recurred 2-3times/day lasting for 1-2 minutes, later generalized tonic clonic seizures were noted during his first two years of life. He was admitted twice to ICU with status epilepticus. His seizures were medically intractable to multiple antiepileptic drugs (Phenobarbitone, Carbamezapine, Topiramate, and Levetiracetam). His development was normal until 5 months of age, then he had a slower gain of milestones, walked at 24 months, climbed upstairs alone at 30 months. He has intellectual disability with severely delayed speech and social milestones, and with no sphincter control. He had normal vision and hearing. He has sleep disturbance, ADHD, and some autistic-like features. Self-mutilation, gratification phenomena, and hyperphagia were other behavioral disorders noted. Melatonin, and Risperidone partially improved his symptoms. Family history revealed intellectual disability and epilepsy in his older brother as well as intellectual disability in three sons of two paternal uncles. Also, there was positive history of congenital heart disease in the family. He has mild dysmorphic features with light hair color, no neurocutaneous stigmata, but with a normal head circumference (52cm at 12 years of age). Cranial nerves were intact with normal tone, power, reflexes, and gait. He had persistent abdominal distension with no organomegaly.  Basic work up including liver function test, and renal profile were normal. Serum ammonia, lactate, amino acids, biotinidase, phytanic acid, and very long chain fatty acids were normal. Urine organic acid analysis was unremarkable. Celiac disease screen was negative. Thyroid function test was normal. Abdominal ultrasound was normal. High resolution karyotype and Fragile X study were negative. Vinland social scale (as he had expressive language disorder) showed IQ of severe ID range (a 2-year performance at 7 years chronological age). EEG initially showed bifrontal epileptiform discharges involving mainly right frontal region with no electrographic seizures, while the brain MRI was unremarkable. Repeated EEG showed a mild intermittent asynchronous with slow activity on right anterior region suggestive of subcortical dysfunction of anterior region. Single photon emission tomogram (Brain SPECT scan) was normal. A follow up EEG showed generalized cortical slowing suggestive of diffuse encephalopathy. Ophthalmological evaluation was normal. Electrocardiogram, and Echocardiogram were normal. He was given a diagnosis of familial ID with epilepsy (partial with 2nd generalization). A follow up brain MRI was also normal. Brainstem auditory evoked response (BAER), electroretinography (ERG) and flash visual evoked potentials (FVEP) were normal bilaterally. |
| 16DG0874 | *ARL6IP1*  NM_015161.1:c.112C>T:p.(Arg38* ) | A 2.5 years old girl with family history of neurological problems. She has born full term to a consanguineous couple. Severe unexplained hypotonia was noted since birth. Examination revealed dolichocephaly, high arched palate, low set years and long tapering fingers. Anterior fontanel was open. She has marked leg, truncal hypotonia and peripheral hypotonia. She has muscle weakness and wasting of the calf muscle, bilateral wrist-drop and claw hand positioning and talipes. MRI brain was normal. One of her siblings died at the age of 6 months in NICU. |
| 17-3865 | *ATP8A2*  NM_016529.4:c.2212-1G>C | A 7-year-old boy born to a consanguineous Jordanian couple. His family sought medical advice when he was 3 years of age because of developmental delay. His evaluation confirmed global developmental delay and revealed the presence of dyskinetic movement. Brain MRI was suggestive of leukodystrophy. EEG was abnormal with multifocal activity. He has two healthy siblings. |
| 15DG2605 | *C12orf4*  NM_ 020374.2:c.1566+1G>T | A 28 months old boy born to consanguineous parents. Pregnancy was complicated by threatened abortion in the first trimester but later progressed uneventfully. Delivery was by emergency C/S due to cephalopelvic disproportion and prior C/S. He had no perinatal complications, was discharged in good condition, and had unremarkable neonatal life. His parents got concerned about his floppiness and development following an episode of diarrhea at the age of 3 months, which has shortly been followed by chest infection requiring admission. After another episode of chest infection at 9 months, he was admitted for further investigation and was found to have GERD. He was evaluated for hypotonia and muscle biopsy was done, revealing non-specific changes. Review of systems was unremarkable, there was no history of seizures and developmental history revealed that sitting was attained at 28 months, and he could say less than 10 words. Parents are first cousins and his older brother is normal. There is history of three cousins suspected to have intellectual disability or autism. Examination showed no dysmorphic features apart from mild epicanthus. Weight was 12.1 kg (slightly below 25th centile), length 86 cm (5-10th centile), and head circumference 49 cm (slightly below the 50th centile). There was hypotonia associated with hypoactive deep tendon reflexes. CT brain was normal |
| 18DG0161 | *CDK9*  NM_001261.3:c.673C>T:p. (Arg225Cys) | An 11 year-old Qatari girl born to consanguineous parents. Pregnancy and delivery were unremarkable. He presented with failure to thrive, global developmental delay and epilepsy with history of choanal atresia. She has facial dysmorphic features including bilateral epicanthic folds, high nasal bridge, multiple bilateral preauricular ear tags, retrognathia, midface hypoplasia and asymmetric face. There is history of ventricular septal defect (closed now), and mild gastro-oesophageal reflux. Eye examination showed no coloboma, however, there was evidence of central vision impairment confirmed by ERG. She has subclinical hypothyroidism. CT scan of the temporal bones showed no middle ear and inner ear defects. Brain MRI showed diffuse atrophic brain lesions, MR Spectroscopy showed reduction of N-Acetyl Aspartate and elevation of lactate peak especially in the basal ganglia. There is positive family history of two twin daughters with similar presentation. In addition, there was history of 6 spontaneous miscarriages. |
| 18DG0162 | *CDK9*  NM_001261.3:c.673C>T:p. (Arg225Cys) | A 12 year-old Qatari girl with multiple congenital anomalies, seizures, bilateral deafness, visual impairment and global developmental delay. CT scan showed bilateral soft tissue choanal atresia. MRI brain showed Dandy-Walker variant, cerebellar atrophy, moderate dilatation of lateral ventricles, reduction in the size of right hippocampus and hypomyelination. She has right dysplastic atrophic kidney. Facial dysmorphism includes small eyes, midface hypoplasia and prominent ears. Echocardiography was normal. X-Ray of the thoracolumbar spine showed segmentation anomaly of the vertebrae at the level of T7 vertebra. Rest of the vertebral body heights and intervertebral disc spaces appeared normal. Accentuated thoracolumbar lordosis was seen. Mild generalized reduction in bone density was noted. Parents are first cousins. She has two healthy sisters and history of 2 miscarriages. Pregnancy was unremarkable. |
| 18DG0165 | *CDK9*  NM_001261.3:c.673C>T:p. (Arg225Cys) | A 20-month-old Qatari girl, diagnosed clinically as CHARGE syndrome with bilateral cataract (operated) with secondary glaucoma, bilateral preauricular tags and unilateral choanal atresia. There is history of feeding problems and two tiny muscular VSDs with left to right shunt. Ultra sound of abdomen and sacrum was normal. Head CT showed right bony choanal atresia and bilateral ossicular anomalies (the middle ears ossicles appear dysmorphic, fused and displaced laterally, nearly attaching to the anterior wall of the middle ear cavity. Anomalous anterior location of the facial nerve descending segment course bilaterally was noted. The vestibules appeared bulbous. No CT evidence of eye coloboma but she did have congenital cataracts. |
| 17-1561 | *CENPF*  NM_016343.3:c.3296delT:p.(Leu1099Argfs*3) | 5-year old boy with global developmental delay, growth retardation and severe microcephaly (OFC -5.5 SD below the mean), dysmorphism (small head, low anterior and posterior hair line, triangular face, prominent nose, high arched palate), hyperextensible joints, atrial septal defect (closed), absent left kidney, and cryptorchidism. MRI brain showed reduced overall white matter volume with borderline pachygyria appearance. Molecular karyotyping is normal. |
| 16-3145 | *CNPY3*  NM_006586.3:c.496-4delAC | A 28 months old female patient presented with  intractable epilepsy, infantile spasm, laryngomalacia, hypotonia, brain atrophy, developmental delay and GERD and she is on NGT feeding. She cannot support and is dysmorphic (hairy forehead, short philtrum, low set ears, micrognathia) and has strabismus. The parents are first cousin once removed. They have another similarly affected daughter (6 months old). |
| 17-5919 | *COL27A1*  NM_032888.2:c.4261-1G>A | 5yr old who presented with global developmental delay and abnormal skeletal findings. He has history of hip dislocation and inguinal hernia. His parents are first cousin and he has a similarly affected brother. Examination revealed dysmorphic facies (prominent forehead, depressed nasal bridge, smooth philtrum, conical teeth and low-set ears), scoliosis and undescended testicles. |
| 17-1984 | *DGAT1*  NM_012079.5:c.1374G>A:p.(Trp458*) | A 9 months old girl who presented with severe failure to thrive, frequent bone fractures, nephrocalcinosis and chronic diarrhea of unknown cause for evaluation. She had significant electrolytes imbalance in the form of hypocalcemia, hypomagnesemia, hypercalcinuria and low alkaline phosphatase level as well as severe osteopenia and multiple long bones fractures in both upper and lower limbs. Hypophosphatasia was suspected but ALP gene sequencing was negative. She had frequent infections including bacterial and systemic fungal infections. She presented with septic shock and died at the age of 11 months. |
| 17-3282 | *DMBX1* NM_147192.2:c.367C>T:p.(Arg123Trp) | A 5 year old girl born to consanguineous parents. At age 2 years she was referred to neurologist with global delayed development (sat at 1 year, walked after 2^nd^ birthday, no speech), no seizures, normal hearing and vision. OFC on the 10th -20 centile (46 cm), marked truncal hypotonia and mild peripheral hypotonia, normal deep tendon reflexes. Brain MRI is unremarkable |
| 17-4220 | *DMXL2*  NM_001174117.1:c.4349_4350insTTACATGA:p.(Glu1450Aspfs*23) | A 36 months old boy, developed focal seizures at age 3 months (admitted for 2 months in PICU). Since then, he was noted to have global developmental delay, no eye contact, moderate degree of cerebral atrophy, macrocephaly and dysmorphic features (long face, high forehead, short philtrum and low set ears). Brain MRI revealed moderate degree of cerebral atrophy including atrophy of the brainstem. Parents are healthy first cousins with no similarly affected children. |
| 17DG0756 | *FUT8*  NM_178155.2:c.943C>T:p.(Arg315*) | A 2 years old boy presented with global developmental delay, dysmorphic features (coarse face with synophrys, hypertelorism, sparse eyelashes, low set ears, thick inverted V-shaped lips, short fingers with clinodactyly, rizomelic shortening). Seizure disorder, failure to thrive, GERD, congenital heart disease (VSD), undescended testis, asthma. Truncal and peripheral hypotonia. Parents are first cousin with family history with three similarly affected children, one of whom passed away at age 2 years. She had two healthy sisters. |
| 17-3434 | *GEMIN4*  NM_015721.2:c.314C>T:p.(Pro105Leu) | This 7-year-old girl was first evaluated by us at age 6 years for global developmental delay. Her birth was normal. Developmental delay was suspected in infancy. At the age of 12 months she was able to sit without support and started walking at the age of 2 years. Speech was significantly delayed and she only speaks with difficulty. Spells (?seizures) started at the age of 2 years for which she was successfully treated with Depakene at the local hospital. Patient has history of bilateral cataracts and she underwent bilateral lens extraction. Family history is significant for consanguinity, and the parents have two other children with similar course with variable severity. Brain MRI revealed small atrophic cerebellum with prominence of the posterior fossa CSF spaces. |
| 17-1853 | *GOLGA2*  NM_004486.4:c.1594_1595insACCG:p.(Arg532Hisfs*21) | An 11-year old girl presented with global developmental delay, microcephaly (head circumference 41.5 cm at 4 yrs and 42.5 at age 11). Best motor milestone achieved was sitting unsupported. She has brisk deep tendon reflexes with clonus. She has unique redness or erythema of the feet and hands secondary to probably autonomic dysfunction. The limbs also are cold on examination. Raynaud's phenomenon was not noted, however. She has profound language delay, vision is unaffected. She has motor weakness affecting lower extremities more than upper ones with joint contractures evolving later with finger flexor contractures, elbow contractures. She has good social/eye contact but has stereotypes with hand clapping and shaking. No seizures. Her clinical course was relatively static with no clear regression. Her CK was elevated.  Muscle biopsy shown dystrophic muscle changes with normal expression of cytoskeletal antibodies for 3 anti-dystrophin antibodies; alpha, beta, gamma and delta sarcoglycans; merosin; the 2 dysferlins, emerin and beta dystroglycan. Brain MRI (at 2-years of age) showed brain atrophy with thin but complete corpus callosum and mild delay and hypomyelination. the posterior fossa structures were unremarkable. MR spectroscopy was unremarkable. |
| 17-1345 | *HCN2*  NM_001194.3:c.1937_1938insT:p.(Met647Hisfs*31) | A 4-year-old girl with global developmental delay and controlled epilepsy. Her abnormal movement started at 1 month of age, occurring during sleep in the form of stiffness of upper and lower limbs and up rolling of the eyes, lasting for 2 minutes.  Has motor and speech delay and so far, cannot make single sentence. Minor dysmorphic features were noted in the form of broad nasal bridge and micrognathia). Normal audiology assessment. Her brain MRI and EEG are unremarkable. Parents are 1st degree cousin. She had 7-year-old similarly affected brother and 3 other healthy siblings. |
| 17DG0987 | *HCRT*  NM_001524.1:c.17_18del:p.(Thr6Lysfs*?) | This patient and two other siblings had an identical presentation with symptom starting early in life with moderate developmental delay, hypotonia, recurrent event of loss of tone mimicking atonic seizures with normal interictal EEG. Later in life these episodes are triggered by emotions such as laughter and fear. They have subclinical central hypothyroidism and normal brain MRI. |
| 17-5538 | *KIAA0556*  NM_015202.2:c.1672C>T:p.(Arg558*) | A 3 years 10 months old Saudi boy, product of full term and normal spontaneous vaginal delivery was noted to be irritable in the neonatal period, and later found to have developmental delay (rolled over at 14m, still cannot sit, no spoken words, unable to hold objects). There is no history of seizures. Parents are first cousin with 4 healthy children. Examination revealed borderline microcephaly (46.6cm on the 5^th^ centile), wt was 10.2 just below 10^th^ centile, postaxial polydactyly in both hands, strabismus vs. oculomotor apraxia, and cryptorchidism. Cardiac echo showed atrioventriculoseptal defect, brain MRI showed mild superior vermis hypoplasia. |
| 17-4991 | *LENG8*  NM_052925.2:c.1981G>A:p.(Glu661Lys) | A 4year old girl who presented with developmental delay, hypotonia since birth, and dysmorphic features (triangular face, brachycephaly epicanthal fold, hypertelorism, broad nasal bridge, and strabismus). Brain MRI shown left middle frontal and bilateral anterior temporal small arachnoid cysts with some adjacent mass effect. Healthy double first cousin parents with no other affected children. |
| 17-1170 | *MCIDAS*  NM_001190787.1:c.717+2T>G | This child presented with respiratory distress and found to have hypoxemia and chest X-ray showed multi-lobar atelectasis with air-trapping. Immunology work up was normal, sweat chloride was normal, follow up chest x ray showed progressive bronchiectasis. Examination revealed short stature. He was also found to have non-obstructive hydrocephalus on imaging. |
| 17-3174 | *MTMR9*  NM_015458.3:c.1415A>T:p.(Asn472Ile) | 11 years old boy with global developmental delay, spasticity, and epilepsy. He was healthy until 4m of age when epilepsy and infantile spasm ensued. Head circumference 52 cm (5th centile) wheelchair-bound, nonverbal, nondysmorphic, no neurocutaneous stigmata . Decreased muscle bulk , spasticity (more in the lower limbs) and brisk deep tendon reflexes. EEG is abnormal. His brain MRI showed bilateral frontoparietal nonspecific T2 hyperintense white matter foci. Parents are first cousins with family history of two affected girls with intellectual disability and 2 healthy kids. |
| 17-0807 | *MYH11*  *NM_022844.2:c.1033+1G>A* | A 27-year-old female G5P1AB3. First pregnancy was spontaneous abortion at 2 months. The second pregnancy was terminated at 15wks due to a massively dilated bladder. The third pregnancy was IUFD at 7 months, delivered vaginally. The fourth pregnancy was terminated due to Potter sequence at 15wks. She presented in the current pregnancy at 16 weeks. Fetal ultrasound documented a massively dilated bladder with echogenic kidney and bilateral hydronephrosis with normal liquor. The patient was offered cordocentesis for whole exome sequencing and DNA banking. The procedure was followed by termination of pregnancy. |
| 17-2355 | *MYO9A*  NM_006901.3:c.1537C>T:p.(Arg513*) | A fetus with no observed movements, and severe ventriculomegaly in both sides with dangling choroid plexus. The rest of the fetal scan was normal |
| 18DG0406 | *PARS2*  *NM_152268.3:c.283G>A:p.(Val95Ile)* | A 34 months old girl referend with microcephaly (HC:35 cm/ at 18 months), severe developmental delay, no contact or interaction, spasticity of all limbs, mild dysmorphism and seizures (mainly myoclonic). MRI brain revealed severe brain atrophy. Her sibling is 7 years 8 months old is also affected with microcephaly (HC: 39 cm/ at 5 YEARS:), severe developmental delay, spasticity of all limbs, failure to thrive and seizures (multifocal)  MRI at 5 years of age showed global cerebral volume loss predominantly within the white matter subcortical and deep component as well as thinning of the overlying cortex resulting in prominence of extra-axial CSF spaces and lateral ventricle. Thinning of the corpus callosum. MR spectroscopy demonstrates mild reduced NAA peak at the basal ganglia and moderate reduced NAA peak at the cerebral cortex in keeping with neuronal loss. Their lactate (serum) and liver enzymes were normal. Parents are first cousins. |
| 17-4086 | *PLOD3*  NM_001084.4:c.1354C>T:p.(Arg452*) | A 9 years old male who presented with failure to thrive, developmental delay and skeletal findings. He had no head control at 6 months, could not sit at 10 months, and only walked at 2 years. He has history of undescended right testicle status post orchidopexy. Examination revealed microcephaly, truncal hypotonia, flat occiput, midface hypoplasia, bilateral ptosis, blue sclera, depressed nasal bridge, micrognathia, low set posteriorly rotated small ears, camptodactyly, and distal arthrogryposis. Parents are distant cousins and he has an affected sister. |
| 17-1682 | *PREPL*  NM_001042385.2:c.1447C>T:p.(Gln483*) | A 9-year-old boy who presented with intellectual disability (no seizures), short stature, chronic lung disease, and failure to thrive. He looked thin, conscious, alert and oriented. There was no history of weakness or fatigue. There was no ptosis and he had normal extraocular movements. The rest of the cranial nerve examination was normal. Motor examination was normal. no weakness and no fatigable weakness. He had 3 affected brother with same condition. Brain MRI was unremarkable. |
| 17-2764 | *QRSL1*  NM_018292.4:c.1042+3A>G | A 9 years old boy with spastic diplegia, cognitive impairment, deafness and episodes of encephalopathy. Brain MRI revealed progressive and extensive abnormal signal involving the deep gray matter structures including the hypothalami, medial aspect of the thalami, basal ganglia and substantia nigra in a symmetric fashion most consistent with Leigh disease. Diffusion restriction in the midbrain lesions suggestive of acute disease. Lactic acid in blood fluctuated between high and high normal. There is positive family history of a similarly affected sister. |
| 18DG0163 | *RUBCN (KIAA0226)*  NM_001145642.2:c.2489delC:p.(Ala830Valfs*146) | A five years old Saudi boy who was delivered by emergency C/S as preterm, gestational age 33 weeks. Mother was 29 years old G5 P2+2A. Parents are first cousins. A15years old sibling suffered from delayed motor and cognitive development and ataxic gait (spinocerebellar ataxia). Examination showed no dysmorphic features, systemic examination showed respiratory distress requiring oxygen via nasal canula, which settled quickly. Neurologic examination was normal, other systems were normal. He was discharged from NICU and followed up in clinic where he was found to have delayed neurodevelopmental milestones, delayed motor function and speech and unsteady gait, he was also found to have sensorineural hearing impairment. Laboratory investigations showed normal electrolytes, renal, hepatic and hematologic functions. Metabolic screen for metabolic disorders was unremarkable. MRI brain at the age of 2 ½ years was reported as normal for age. |
| 17DG1005 | *SCLT1*  NM_144643.2:c.290+2T>C:p.(Lys79Valfs*4) | Two siblings were diagnosed with congenital panhypopituitarism. They have the same phenotypic and hormonal deficiency affecting only GH and Thyroid and same radiological abnormalities (hypoplastic anterior pituitary with shallow sella turcica ectopic posterior lobe, and hypothalamic hamartoma). No history of developmental delay or syndromic features on evaluation. |
| 17-2246 | *SLC25A42*  NM_178526.4:c.871A>G:p.(Asn291Asp) | A 15-year-old female with recurrent episodes of ketosis, and lactic acidosis usually triggered by intercurrent illness. There was no history of hyperammonemia or hypoglycemia. The frequency of the metabolic attacks is lessening with age. Urine organic acid analysis and acylcarnitine analysis were normal. Parents are consanguineous. |
| 17-1731 | *SLC9A1*  NM_003047.4:c.938G>A:p.(Gly313Glu) | A 9-year old girl with a history of ataxia and developmental delay. Her course has been static with slow improvement. She is ambulatory with recurrent falls. She has also cognitive delay particularly in language and learning. There is no history of seizures and no history of regression. Her brain MRI showed (at age 6) moderate cerebellar atrophy. MR spectroscopy was unremarkable. She underwent comprehensive metabolic testing which included CBC, renal profile, hepatic profile, lipid profile, transferrin, isoelectric focusing, CK, alpha fetoprotein, very long chain fatty acids, lactic acid, ammonia, tandem MS, biotinidase as well as urine organic acid and creatinine panel, all of which were normal. Her clinical exam is notable for axial and appendicular ataxia, and joint hyperlaxity. There were no pyramidal signs, dysmorphic features or nystagmus. |
| 17-5433 | *STX3*  NM_001178040.1:c.675+1delinsT | A 6 years old girl was born vaginally with unremarkable pregnancy. She presented with developmental delay, hypotonia, and nystagmus. She has history of neonatal jaundice in the first week of her life. |
| 15DG1600 | *TBXT*  NM_003181.3:c.466G>T:p.(Gly156Cys) | A 2.8years Saudi girl was delivered normally at term. Mother was 26 years old G2P1+0. Antenatal U/S at 26 weeks gestation showed small head <5^th^ centile, banana shaped cerebellum, and a mass at the lumbar spine. Examination showed no facial dysmorphic features. There was dorsolumbar myelomeningeocele (3x5 cm), leaking CSF, paraplegia with no movements in the lower limbs which were held in flexion at hips and extended knees, patulous anus with absent anal reflex. Sensations and reflexes were absent in the lower limbs. CT brain revealed hydrocephalus and Chiari malformation with prominent posterior fossa structures and herniated cerebellar vermis. MRI brain showed dilated lateral ventricles, the cerebellum is normally shaped, and it is low lying, with herniation of the vermis, and Arnold-Chiari malformation. The myelomeningocele was repaired, and she required ventriculoperitoneal shunt for subsequent hydrocephalus. She was incontinent of stools and urine. She developed urinary tract infections and sepsis with multidrug resistant klebsiella pneumonae. She was on ventilatory support, she also had refractory seizures. She died at the age of two years. Parents were first cousins and there was strong family history of neural tube defects. Her younger sister who also had neural tube defects and died at the age of 9 months with severe bronchiolitis causing respiratory failure. |
| 17-1906 | *TFG*  NM_001007565.2:c.320G>C:p.(Arg107Pro) | A 4-year- old girl, product of FT SVD. She sat at 6 months of age, crawled at 8 months, she is still unable to walk independently, and is only able to say few words which were not clear. Her vision and hearing were normal. She can recognize her family members. Not dysmorphic but she has spasticity of the lower limbs. The impression when she was at the age of 2 years was spastic paraplegia. Parents are consanguineous related as first cousins and reported to have good health. They have three daughters; two of them are affected with the same presentation. Positive family history of similar illness in her cousin. She also has one uncle and one aunt from her paternal side with similar presentation. |
| 15DG1395 | *THUMPD1*  NM_017736.3:c.706C>T:p.(Gln236*) | A 23 years old male with intellectual disability, dysmorphic features, hypertension and diabetes. He was delivered at term via SVD. Antenatal history was significant for polyhydramnios. Parents are first degree cousins with a similarly affected child. His physical examination showed relatively large skull, sparse hair, right eye exotropic strabismus, up-slanting of palpable fissures, as well as prognathism with mild limitation of jaw opening, hands with tapering fingers, generalized thickening of skin with scattered trunk ale acne. Ophthalmology examination showed right large angle esotropia with hypertropia, and restricted eye movements in all directions except medially. Neurologically he is oriented but with low tone voice and slow response, increased muscle tone of cogwheel type, and slow initiation of gait. |
| 17-3959 | *TXNRD2*  NM_006440.4:c.1321C>T:p.? /ENST00000400525:exon14:c.1252C>T:p.R418X) | A 12 years old Saudi boy, admitted with history of recurrent hypoglycemia, polycythemia.  He has history of congenital heart disease (truncus arteriosus, and pulmonary stenosis). Examination revealed dysmorphic facies (hypertelorism, synophrys, bitemporal narrowing, bulbous nose, thick lips, short neck, cleft palate, large omphalocele, and clubbing of fingers). He was also diagnosed with glucocorticoid deficiency. |
| 17-8378 | *WDR81* NM_001163809.1:c.850_851del:p.(Leu284Valfs*9) | A 35 y.o Saudi lady was seen in the genetic counseling clinic because of two children who died with severe hydrocephalus. Husband is her first cousin. They have 2 healthy children. |
| 17-2310 | *WIPI2*  NM_001033520.1:c.21delC:p.(Phe8Leufs*2) | A 7 month old Sudanese baby girl, product of vaginal delivery, cried immediately after delivery and was discharged home in good condition. She was admitted at age 2 weeks due to suspected tyrosinemia (high tyrosine on newborn screening) with prolonged coagulation profile but gene sequencing and the succinylacetone in urine were negative. Organic acids were suggestive of mitochondrial disease. She developed hypotonia, nystagmus, and jaundice with elevated liver enzymes and alpha-fetal protein. She is the first child to her consanguineous parents. |
| 18DG0164 | *XRCC2*  NM_005431.1:c.643C>T:p.(Arg215*) | A 3years old, Saudi girl, born at full term via vaginal delivery. At birth, she was noted to have borderline microcephaly (OFC was 33 cm), birth weight was 2.4, and length was 48 cm. She was also noted to have bilateral hypoplastic thumb, multiple café late spots, strabismus, and dysmorphic facial features (epicanthal fold and short neck). She developed failure to thrive with progressive microcephaly (at age of three years her weight was 10.4 kg, head circumference was 45 cm, and height was 84.6 cm). Her development was appropriate for her age.  Her parents are second cousins. She has a sister with hypoplastic thumbs, imperforate anus and no cafe au lait spots who died of respiratory failure and pancytopenia.  Investigation showed pancytopenia. Her tandem MS and urine for organic acid were normal. Chromosomal breakage was 100%. Brain MRI at 3 months of age was unremarkable. Right hand MRI revealed bilateral absence of flexor pollicis brevis muscle. Otherwise, the other tendons and bones are present with no abnormality seen. Skeletal survey showed hypoplastic thumb, and mild bilateral acetabular dysplasia. |
| 14DG0265 | *ZAK* NM_016653.2:c.748C>T:p.(Arg250Trp) | 3yr old girl with unexplained myopathy and failure to thrive. She was born following an uneventful pregnancy. Neonatal history was remarkable for poor sucking. Initially, the main concern about her health was regarding her poor weight gain and she was labeled by her physician as failure to thrive when all growth parameters were presumably below the 3rd centile. Later, it became apparent that she had motor delays. She only took independent steps on a flat service at 28m after intensive physical therapy, she was still unable to make intelligible words. Of note, the muscle weakness, while generalized, also involves the neck to the point that she struggles to keep her head straight as it tends to fall forward as if she is unable to support it with her muscles. The pastmedical history is largely negative otherwise and the review of systems did not reveal any salient symptoms such as abnormal movements or seizures. Family history is remarkable for parents being consanguineous and a paternal uncle with unexplained myopathy. CK was never grossly elevated. Muscle biopsy revealed findings suggestive of congenital myopathy. Her brain MRI was was normal. Her thyroid function test was also normal. |
